# Reduced Hyperpolarization-Activated Current Contributes to Enhanced Intrinsic Excitability in Cultured Hippocampal Neurons from PrP^−/−^ Mice

**DOI:** 10.3389/fncel.2016.00074

**Published:** 2016-03-24

**Authors:** Jing Fan, Patrick L. Stemkowski, Maria A. Gandini, Stefanie A. Black, Zizhen Zhang, Ivana A. Souza, Lina Chen, Gerald W. Zamponi

**Affiliations:** Department of Physiology and Pharmacology, Hotchkiss Brain Institute, Cumming School of Medicine, University of CalgaryCalgary, AB, Canada

**Keywords:** cellular prion protein, HCN, excitability, *I*_h_, hippocampus

## Abstract

Genetic ablation of cellular prion protein (PrP^C^) has been linked to increased neuronal excitability and synaptic activity in the hippocampus. We have previously shown that synaptic activity in hippocampi of PrP-null mice is increased due to enhanced *N*-methyl-D-aspartate receptor (NMDAR) function. Here, we focused on the effect of *PRNP* gene knock-out (KO) on intrinsic neuronal excitability, and in particular, the underlying ionic mechanism in hippocampal neurons cultured from P0 mouse pups. We found that the absence of PrP^C^ profoundly affected the firing properties of cultured hippocampal neurons in the presence of synaptic blockers. The membrane impedance was greater in PrP-null neurons, and this difference was abolished by the hyperpolarization-activated cyclic nucleotide-gated (HCN) channel blocker ZD7288 (100 μM). HCN channel activity appeared to be functionally regulated by PrP^C^. The amplitude of voltage sag, a characteristic of activating HCN channel current (*I*_h_), was decreased in null mice. Moreover, *I*_h_ peak current was reduced, along with a hyperpolarizing shift in activation gating and slower kinetics. However, neither HCN1 nor HCN2 formed a biochemical complex with PrP^C^. These results suggest that the absence of PrP downregulates the activity of HCN channels through activation of a cell signaling pathway rather than through direct interactions. This in turn contributes to an increase in membrane impedance to potentiate neuronal excitability.

## Introduction

Cellular prion protein (PrP^C^) is a naturally occurring protein, whose abnormal conformation can lead to a range of neurological disorders including Creutzfeldt-Jakob disease, Kuru and bovine spongiform encephalitis (Knight and Will, 2004). This abnormal protein accumulates in the brain, forming polymeric aggregates that disrupt synapses, leading to loss of dendrites and finally neuronal death (Soto and Satani, 2011). Although this pathological role of misfolded PrP^C^ has been established and studied extensively, the function of PrP^C^ in the normal brain remains largely unknown (Linden et al., 2008; Zamponi and Stys, 2009; Stys et al., 2012; Black et al., 2014). Initial studies with PrP-null mice revealed a mild pathology, such as a slight impairment of spatial learning (Collinge et al., 1994; Manson et al., 1995), but otherwise normal development (Büeler et al., 1992; Manson et al., 1994; Criado et al., 2005). However, several lines of evidence show that PrP-null hippocampal neurons display enhanced susceptibility to cell death induced by serum deprivation (Kuwahara et al., 1999; Haigh et al., 2009), and in response to glutamate excitotoxicity (Khosravani et al., 2008), together indicating a central role of PrP^C^ in neuroprotection. Growing evidence supports the idea that the loss of PrP^C^ function interferes with normal neuronal activity, and therefore contributing to synchronized activities that underlie neocortical and hippocampal seizures (Walz et al., 2002). More specifically, recordings from PrP-null neurons *in vitro* showed a variety of electrophysiological abnormalities, with reduced Ca^2+^-dependent K^+^ currents (Colling et al., 1996), abnormal type-A γ-aminobutyric acid receptor (GABAAR) activity (Collinge et al., 1994), as well as a significant reduction of afterhyperpolarization potentials (AHP; Colling et al., 1996; Herms et al., 2001; Mallucci et al., 2002; Fuhrmann et al., 2006). Despite this progress, we do not yet fully understand how PrP^C^ regulates neuronal output.

In a previous study, we reported that PrP-null mice display enhanced synaptic activity that then gives rise to increased network excitability due to augmented *N*-methyl-D-aspartate receptor (NMDAR) function (Khosravani et al., 2008). Here, we focused on the ability of PrP^C^ to regulate intrinsic neuronal firing properties in hippocampal neurons cultured from P0 mouse pups. We hypothesized that putative differences in membrane excitability can be attributed to specific ionic mechanisms which drive neuronal cell electrical activity. Among various ion channels, the hyperpolarization-activated cyclic nucleotide-gated (HCN1) and HCN2 channels are of particular interest. First, they are highly expressed in the central nervous system and are critically related to neuronal excitability at both single-cell and network levels, particularly in the hippocampus (Robinson and Siegelbaum, 2003). Second, changes in HCN channels have been implicated in animal models of neurological disorders such as pain and epilepsy (Biel et al., 2009; Noam et al., 2011). We found that intrinsic excitability was enhanced in PrP-null hippocampal neurons, which, based on cell morphology, encompassed the features of pyramidal cells. This could, at least in part, be attributed to an increased input resistance and a related down-regulation in HCN channel activity in hippocampal cultures from P0 mice, but curiously not in adult mouse hippocampal slices. These findings indicate that functional regulation of HCN channels by PrP^C^ might have an important role in maintaining normal electrical signals in the brain.

## Materials and Methods

### Primary Hippocampal Culture Preparation

As previously described, mice used in these experiments were wild-type (WT) C57BL/6 mice and a PrP knock-out (KO) Zuerich 1 strain outbred to a pure C57 genetic background by Dr. Frank Jirik’s laboratory (University of Calgary, Canada) (Khosravani et al., 2008; You et al., 2012). Primary hippocampal cells were obtained from P0-P1 pups as described by us previously (Khosravani et al., 2005; You et al., 2012). Cells were maintained in culture for 10–13 days (DIV 10–13) before experimentation. All procedures were approved by the University of Calgary animal care committee.

### Immunoblots and Co-Immunoprecipitations (co-IPs)

HCN1 and HCN2 protein levels were measured using a standard protocol as described by us previously (Khosravani et al., 2008). Two different protein loading amounts (100 and 150 μg) were used in each condition in order to verify a linear range of signal detection. Membranes were probed with mouse anti-HCN1 (1:500, Neuromab, 75-110) or mouse anti-HCN2 (1:500, Neuromab, 75-111). For co-IPs, mouse hippocampi were homogenized in cold lysis buffer with a protease inhibitor and treated with 1 μM CuSO_4_, and co-IPs between HCN1 or HCN2 and PrP^C^ were performed as described by us previously (Khosravani et al., 2008). Complexes were precipitated with the mouse anti-PrP 6H4 antibody (Prionics USA), and immunoblots were probed with mouse anti-HCN1 or mouse anti-HCN2 antibodies (1:500, Neuromab).

### Electrophysiology

Whole-cell voltage and current clamp recordings from cultured neurons were performed in a bath solution containing (mM) 150 NaCl, 5 KCl, 2.5 CaCl_2_, 1 MgCl_2_, 10 HEPES and 10 D-glucose (adjusted to pH 7.4 with NaOH). The intracellular pipettes were pulled from borosilicate glass (with an impedance of 3–5 MΩ) and filled with an intracellular solution containing (mM) 130 KGluconate, 4 Mg-ATP, 0.3 Na-GTP, 10 EGTA, 2 CaCl_2_ and 10 HEPES (adjusted to pH 7.2 with KOH; osmolarity 310–320 mOsm). Whole-cell recordings were made from hippocampal cultures at room temperature using an Axopatch 200B amplifier (Axon Instruments) with Clampex 9.2 software running on a computer to acquire data. Neurons were placed in bath solutions for 15–30 min prior to recordings. Electrical activity was assessed in current- and voltage-clamp configurations. Current-clamp recordings from neurons that had membrane potentials more negative than −50 mV were included in the analysis. The holding membrane potential was manually adjusted to −70 mV. During recordings, the command potential was monitored and compensated for the voltage drop across the electrode. The injected currents for compensation were less than 100 pA. Series resistance in voltage-clamp recordings was compensated by 50–70% and continually monitored through experiments. Recordings were terminated whenever significant increases (≥20%) in access resistance occurred. Voltage and current signals were filtered at 2 kHz and sampled at 5 kHz and 20 kHz (Digidata 1320A, Molecular Devices), respectively, in all experiments. Slice recordings from adult hippocampal slices were prepared as described by us previously (Khosravani et al., 2008) in the presence of synaptic blockers.

#### Current-Clamp recordings

To investigate the firing patterns of neurons, 250 ms depolarizing current steps from −200 pA in increments of 50 pA were applied from a holding potential of −70 mV. The latency of each action potential (AP; cumulative latency) was measured in response to 0.7 nA, 500 ms depolarizing current ramps for the greatest number of both type of neurons to fire APs. APs were generated with 5 ms depolarizing current pulses. Electrical membrane properties, including resting membrane potential (*E*_m_), AP_amplitude_, AP duration at 50% amplitude (AP half-width), maximum rate of depolarization (*dV/dt*_max_), and maximum rate of repolarization (*rV/rt*_max_) were analyzed. The AP threshold was measured at rest by off-line differentiation of voltage traces, thereby determining the first point on the rising phase of an AP where the rising rate exceeds 50 mV/ms (Stemkowski and Smith, 2012). In response to a −350 pA hyperpolarizing pulse with 800 ms duration, voltage sag amplitude was measured. Percentage sag was calculated using the equation:

$$\text{Sag}\;\text{ratio}\;=\;\left(\frac{V_{\text{peak}}-V_{\text{ss}}}{V_{\text{peak}}}\right)\times 100,$$

where *V*_peak_ is the maximum voltage deflection and *V*_ss_ is the steady state voltage at the end of the hyperpolarizing pulse (George et al., 2009).

#### Voltage-Clamp Recordings

Input resistance (Rin) was determined by a brief 20 mV hyperpolarizing pulse (from −70 mV to −90 mV, 40 ms duration) and reflected by a steady-state current. *I*_h_ was recorded in external and internal solutions that were the same as those used for AP recordings. *I*_h_ was evoked by a series of hyperpolarizing voltage steps from −50 mV to −150 mV in increments of 10 mV and also in decreasing durations from 4 s to 2 s. The amplitude of *I*_h_ was measured as the difference between the steady-state current at the end of each test potential and the instantaneous current immediately following each test potential. Under these recording conditions, the presence of *I*_h_ could be confirmed by susceptibility to the specific HCN channel blocker ZD7288. The kinetics of *I*_h_ activation were best determined by fitting onset of the current with a single-exponential function: $f(t)\;=\;Ae\left(\frac{t}{\tau }\right)+C$ (Han et al., 2011; Bonin et al., 2013). Additionally, to assess voltage-sensitivity of *I*_h_, the half-maximal activation (*V*_0.5_) was determined by fitting individual conductance-voltage (G-V) relationships with a Boltzmann function:

$$\frac{G}{G_{\max}}\;=\;\frac{1}{\left\{1+\exp\left[\frac{\left(-V_{0.5}-V_{m}\right)}{k}\right]\right\}},$$

where *G*_max_ is the mean value of fit maximal conductance, *V*_0.5_ is the membrane potential for the half-activation and *k* is the slope factor. The conductance was calculated according to the equation: *G* = *I*_h_/(*V*_m_ − *E*_h_), where *G* is the conductance, *I*_h_ is the HCN tail current, *V*_m_ is the holding potential, and *E*_h_ is the reversal potential of *I*_h_. The reversal potential of *I*_h_ was determined to be −46 ± 2 mV by linear extrapolation to the peak of the tail current from clamping at potentials between −40 and −80 mV.

### Drugs and Drug Application

In all recordings, the ionotropic glutamate antagonists _D, L_-APV (50 μM) and DNQX (20 μM) were added to the external solution. In voltage-clamp recordings, tetrodotoxin (TTX, 1 μM) and BaCl_2_ (0.5 mM) were added to the external bath to improve isolation of *I*_h_. Forskolin (20 μM) was bath applied for pharmacological studies of HCN channels. ZD7288, DNQX, _D, L_-APV and TTX were acquired from Tocris Bioscience. Other chemicals were from Sigma.

### Data Arrangement and Statistical Analysis

Statistical analyses were performed using Origin9 and Sigmaplot10.0. The cumulative latency analysis was conducted through the seventh current ramp step (0.7 nA) in order to reach the maximal numbers of spikes in both groups. Data are expressed as means ± SEM. Statistical analyses were done using two-tailed unpaired Student’s *t*-tests. Significance was set at *p* < 0.05.

## Results

### Membrane Properties and Enhanced Intrinsic Excitability in Cultured PrP-Null Neurons

Our previous studies demonstrated that mice lacking PrP^C^ display increased synaptic activity in part due to enhancement of NMDAR function (Khosravani et al., 2008). Here, we focused on intrinsic excitability of hippocampal neurons by examining the firing pattern and membrane properties of hippocampal neurons from PrP-null mice.

**Figure 1:**
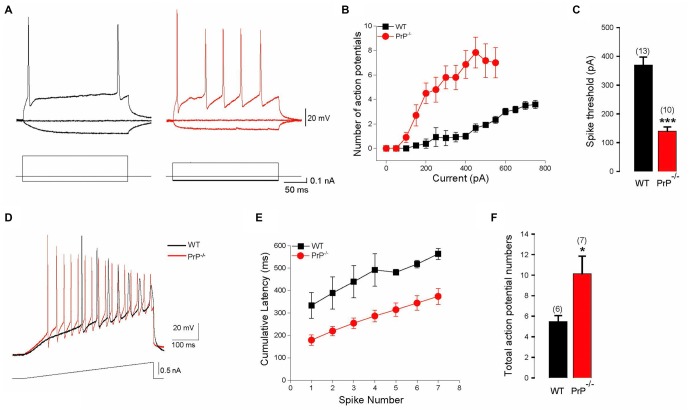
**Enhanced intrinsic excitability in cultured prion protein (PrP)-null neurons. (A)** Representative action potentials (APs) evoked by depolarizing pulses in a hippocampal neuron cultured from wild-type (WT; black) and PrP-null mice (red). **(B)** Average number of APs induced by increasing depolarizing currents in cultured neurons from WT (*n* = 13) and PrP-null mice (*n* = 10). **(C)** Average spike threshold for WT and PrP-null neurons. **(D)** APs evoked by a 500 ms depolarizing current ramp for WT (black) and PrP-null (red) neuron. **(E)** Cumulative AP latencies for WT and PrP-null neurons (*p* < 0.001 for the induced AP from the first to the seventh). **(F)** Average number of APs during current ramp application for WT (*n* = 6) and knock-out (KO) neurons (*n* = 7). *p < 0.05, ***p < 0.001.

We used intracellular recordings from cultured hippocampal pyramidal neurons to characterize the effect of PrP^C^ on hippocampal neuron intrinsic excitability in the presence of synaptic blockers DNQX (20 μM) and _D, L_-APV (50 μM). We found that the absence of PrP^C^ strongly affected firing properties of hippocampal neurons, increasing number of APs (Figures 1A,B) and decreasing the spike threshold (Figure 1C) in response to 250 ms depolarizing step current injections (369.2 ± 28.6 pA for WT (*n* = 13) vs. 140.0 ± 14.5 pA for KO (*n* = 10), *p* < 0.001). Further alterations in membrane excitability were determined through measurements of AP latency and the total number of APs generated in response to depolarizing current ramps (steps from 0.1 nA to 1.0 nA in increments of 0.1 nA). All analysis was conducted on ramps up to 0.7 nA, because responses saturated at higher stimulation intensities (Figures 1D–F). Significant reductions in cumulative AP latencies (Figure 1E), as well as a significant increase in total AP number were observed (5.5 ± 0.6 APs, *n* = 6 for WT vs. 10.1 ± 1.7 APs, *n* = 7 for KO, Figure 1F, *p* < 0.05). This is further suggestive of increased excitability in neurons lacking PrP^C^, and is consistent with what was previously found in slice recordings (Colling et al., 1996). In addition, a significant increase in input resistance was observed in PrP-null neurons (256 ± 35.6 MΩ for WT (*n* = 8) vs. 361 ± 24.8 MΩ for KO (*n* = 11), Table 1, *p* < 0.05). However, there was no significant difference in the resting membrane potential or in the AP characteristics between WT and null neurons (Table 1). Taken together, higher input resistance may partially account for the hyperexcitability observed in PrP-null neurons.

**Table 1:** **Electrophysiological properties of hippocampal neurons in wild type (WT) and prion protein (PrP)-null mice**.

	*E*_m_ (mV)	*R*_in_ (MΩ) (mV)	AP_amplitude_ (ms)	AP_half-width_ (mV/ms)	*dV/dt*_max_ (mV/ms)	*rV/rt*_max_ (mV)	Threshold
WT	−55 ± 1.4 (5)	256 ± 35.6 (8)	107 ± 6.6 (5)	1.9 ± 0.2 (5)	132 ± 23.5 (5)	−67 ± 6.2 (5)	−42 ± 2.0 (5)
PrP^−/−^	−54 ± 1.3 (9)	361 ± 24.8 (11)	116 ± 2.3 (9)	1.8 ± 0.1 (9)	136 ± 13.8 (9)	−76 ± 7.9 (9)	−39 ± 2.6 (9)

*Electrophysiological parameters were measured using whole-cell voltage clamp and current clamp recordings as described in “Materials and Methods” Section. Numbers of cells testes are shown in parentheses*.

We also carried out experiments in acute hippocampal slices from ~2 month WT and PrP-null mice. Interestingly, under these conditions, we did not observe differences in input resistance (107.5 ± 5.8 MΩ for WT (*n* = 6) vs. 96.0 ± 4.6 MΩ for KO (*n* = 7), *p* > 0.05). There was no difference in the numbers of action potentials evoked over a wide range of current injections (from 150 pA to 650 pA), and there was no difference in spike threshold (210.1 ± 43.0 pA for WT (*n* = 5) vs. 207.1 ± 17 pA for KO (*n* = 7)). Only cells with a leak smaller than 20 pA were included in this analysis. There was, however, a statistically significant decrease in voltage sag ratio (0.11 ± 0.01 for WT (*n* = 10) vs. 0.07 ± 0.01 for KO (*n* = 10), *p* < 0.01). While this latter observation generally fits with our findings in cultured neurons, this change does not appear to be sufficient to drive a statistically significant difference in the intrinsic excitability of the neurons in the slice preparation. Alternatively, it is possible that there are changes in other conductances that may compensate for the effects of the voltage sag in slices from these older animals compared to the cultured neurons.

### Reduced *I*_h_ and Down-Regulated HCN Channel Functional Properties in Hippocampal Cultures from PrP-Null Mice

HCN channels are major determinant of input resistance (Yamada-Hanff and Bean, 2015). Given the role of these channels as a physiological voltage clamp, we determined whether the observed biophysical changes are related to alterations in HCN channel function.

**Figure 2:**
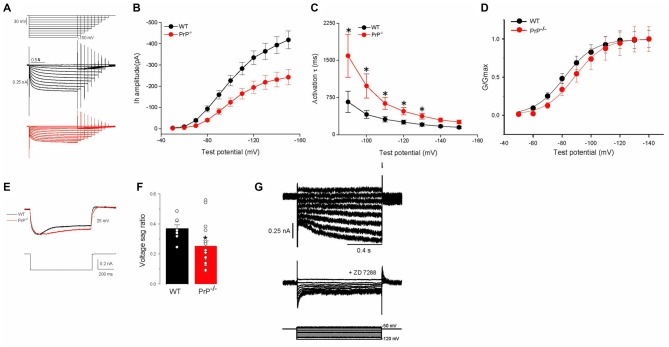
**Characteristics of *I*_h_ in wild type (WT) and PrP-null neurons. (A)** Evoked responses from WT and PrP-null neurons to hyperpolarizing voltage steps from −50 mV to −150 mV in increments of 10 mV and also in decreasing durations from 4 s to 2 s in the presence of Ba^2+^ (0.5 mM) and TTX (0.5 μM). **(B,C)** Summary of *I*_h_ amplitude and activation time constant (τ). *I*_h_ amplitude was significantly smaller (*n* = 24 for WT, *n* = 20 for KO) and kinetics were slower in PrP-null neurons (*n* = 11 for WT, *n* = 9 for KO). **(D)** Normalized HCN channel activation curves in PrP-null neurons showed a hyperpolarizing shift in half activation voltage (*n* = 22 for WT, *n* = 17 for KO). **(E)** Sample recordings showing a voltage sag generated by *I*_h_ in response to the hyperpolarizing current step at −300 pA. PrP-null neurons displayed a decrease in the amplitude of the sag response. **(F)** Summary of the voltage sag ratio between WT (*n* = 8) and PrP-null neurons (*n* = 16). **(G)** Family of current records illustrating the effect of ZD7288 (100 μM) on *I*_h_ in WT neurons. The pulse protocol used for this experiment is depicted at the bottom.

*I*_h_ was induced by a series of hyperpolarizing voltage steps (Figure 2A). To improve stability in recordings, step incrementally decreased in duration from 4 s to 2 s. The maximal amplitude of peak current measured following the stimulation was decreased in PrP-null neurons (*V*_cmd_ = −150 mV: peak current 418.2 ± 41.7 pA for WT (*n* = 24) vs. 242.6 ± 35.5 pA for KO (*n* = 20), Figure 2B, *p* < 0.01). In addition, a voltage-independent component in the currents that incorporated HCN channels was activated instantaneously (*I*_inst_) (Proenza et al., 2002; Proenza and Yellen, 2006). The amplitude of this instantaneous current in PrP-null neurons was also significantly downregulated (data not shown). To determine whether the decrease in *I*_h_ in PrP-null neurons resulted from alterations in the kinetics of *I*_h_ activation, we fitted the onset of the current with a single-exponential function. As shown in Figure 2C, the time constant was decreased at more negative voltages in both WT and PrP-null neurons. However, the time course became significantly slower in PrP-null neurons over the command voltage range compared to WT neurons (*p* < 0.05 at steps between −90 to −130 mV). A shift in the voltage dependence of *I*_h_ activation may also explain the reduction in *I*_h_, and therefore the lack of PrP^C^ on steady-state activation was determined by fitting normalized conductance to a Boltzmann function. This revealed that the *V*_0.5_ value was significantly shifted to a more hyperpolarized level in PrP-null neurons (*V*_0.5_: −80.82 ± 2.16 mV for WT (*n* = 22) vs. −89.86 ± 1.95 mV for KO (*n* = 17), Figure 2D, *p* < 0.05). However the slope factor was not altered (10.9 ± 0.7 mV for WT (*n* = 22) vs. 11.6 ± 0.9 mV for KO (*n* = 17), *p* > 0.05). Consistent with the changes observed for *I*_h_, current-clamp recordings reveal that the amplitude of the voltage sag, a characteristic of *I*_h_ activation, was decreased in PrP-null neurons (sag ratio: 0.37 ± 0.03 for WT (*n* = 8) vs. 0.25 ± 0.04 for KO (*n* = 16), Figures 2E,F, *p* < 0.05). *I*_h_ and voltage sag recorded from WT and null neurons were completely blocked by the HCN blocker ZD7288 (100 μM; see Figure 2G for a sample recording from WT neuron), indicating that the measured current including the instantaneous phase was indeed mediated by HCN channels. These results suggest that PrP^C^ functionally regulates the biophysical properties of HCN channels.

### The Difference in Input Resistance is Eliminated by HCN Blockers

**Figure 3:**
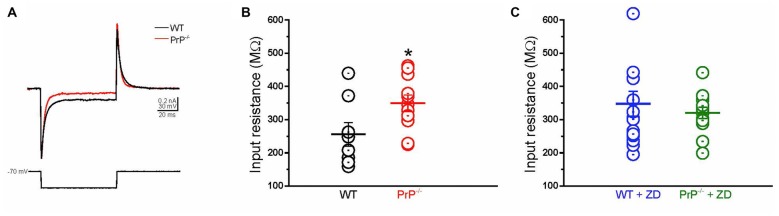
**Enhanced input resistance in cultured PrP-null neurons. (A)** Sample currents recorded from WT (black) and PrP-null neurons (red) in response to a short hyperpolarizing voltage step from −70 mV to −90 mV. **(B)** Input resistance of PrP-null neurons (*n* = 11) was 29.2% higher than input resistance of WT (*n* = 8). **(C)** There was no distinguishable difference in the input resistance of WT (*n* = 15) and PrP-null neurons (*n* = 13) in the presence of ZD7288. *p < 0.05.

As HCN channels are partially active at rest, activation of *I*_h_ depolarizes the membrane potential and reduces membrane impedance (Wang et al., 2007). To explore whether the enhancement in input resistance in PrP-null neurons resulted from the decrease in *I*_h_, we examined the effect of the HCN channel blockade (Figures 3A,B). In WT neurons, inhibition of *I*_h_ by ZD7288 resulted in an increase in input resistance (*p* < 0.05, compare panels B and C), which is consistent with a previous study (Lupica et al., 2001; Aponte et al., 2006). It is worth noting that the activation of *I*_h_ in PrP-null neurons was negligible at −70 mV (Figure 2D) where input resistance was measured. Therefore, the input resistance tested in PrP-null neurons before and after ZD7288 applied was similar (*p* > 0.05). Notably, the input resistance in WT and PrP-null neurons became indistinguishable in the presence of ZD7288 (Figure 3C, *p* = 0.5), providing evidence for a mechanistic link between PrP^C^ and HCN channel activity.

### Exploration of HCN Subunit Composition and Physical Interaction with PrP^C^

**Figure 4:**
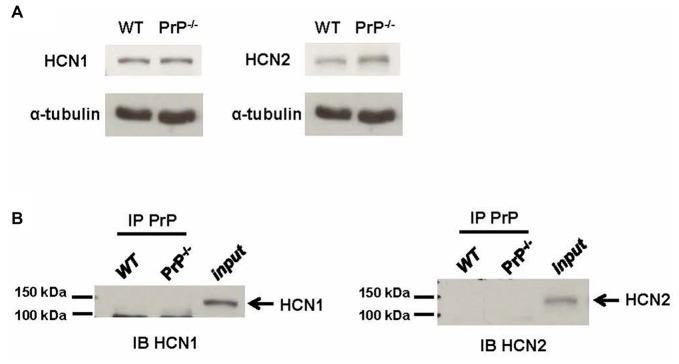
**Analysis of hyperpolarization-activated cyclic nucleotide-gated (HCN) subunit expression. (A)** Representative immunoblots for HCN1 (left) and HCN2 subunit (right) proteins in membrane homogenates prepared from hippocampi of WT and PrP-null adult mice. α-tubulin was used as a loading control for each sample. This experiment is representative of three different repetitions (protein expression normalized to α-tubulin for HCN1: 1.24 ± 0.07 for WT vs. 1.29 ± 0.06 for KO, *p* > 0.05; for HCN2: 1.47 ± 0.48 for WT vs. 1.41 ± 0.07 for KO, *p* > 0.05). **(B)** Co-Immunoprecipitations (co-IPs) of PrP^C^ and HCN subunits using lysates from WT and PrP-null adult mice hippocampal tissue showing that neither HCN1 nor HCN2 associated with PrP^C^. The lanes for a co-IP control (IP PrP^C^) from PrP-null appeared to be blank which was also observed in the lanes for a bead-only control (data not shown). HCN1 and HCN2 protein could both be detected in hippocampal homogenates (input). This experiment was repeated three times with identical results.

HCN subunit composition determines the functional properties of the channel, including cyclic adenosine monophosphate (cAMP) sensitivity, voltage-dependent activation and kinetics, as well as interactions with intracellular signaling pathways (He et al., 2014). We thus wished to explore whether the observed changes in HCN function were mediated by an HCN subunit switch. Antibodies against HCN1 or HCN2 subunits, the principal HCN subunits expressed in the hippocampus, were used in a standard immunoblotting procedure. To obtain sufficient material for these biochemical measurements, we used hippocampal homogenate from adult mice rather than cultured neurons. These experiments revealed that the expression of HCN1 and HCN2 subunits detected from hippocampal homogenates was similar in WT and null neurons (Figure 4A; relative protein expression of HCN1: 1.24 ± 0.07 for WT (*n* = 3) vs. 1.29 ± 0.06 for KO (*n* = 3), *p* > 0.05; relative protein expression of HCN2: 1.47 ± 0.48 for WT (*n* = 3) vs. 1.41 ± 0.07 for KO (*n* = 3), *p* > 0.05). We also examined whether cell surface expression of HCN1 and HCN2 channels was altered in PrP null neurons, however, no statistically significant difference in plasma membrane protein level was observed (*n* = 6, data not shown).

It is also possible that PrP^C^ mediated changes in HCN channel function may arise from a direct association of PrP^C^ with the channel. To explore this possibility, we performed co-IPs from WT mouse hippocampal homogenate. However, neither HCN1 nor HCN2 could be co-immunoprecipitated with PrP^C^ (Figure 4B). Given that PrP^C^ function is copper-dependent (Stys et al., 2012; Black et al., 2014), the negative results from co-IP could potentially be due to the absence of copper ions in our solutions. Hence, the co-IP experiments were also repeated in the presence of a high concentration of 1 μM copper, which is similar to the resting copper concentration in the synaptic cleft (Millhauser, 2007; Stys et al., 2012). However, the results remained unchanged (data not shown). Taken together, these data indicate that the effect of PrP^C^ on HCN channel function is not due to alterations in HCN subunit expression, nor due to a direct physical effect of PrP^C^ as part of a molecular complex.

### A PrP^C^-Mediated cAMP-Dependent Pathway Regulates HCN Channel Activity

**Figure 5:**
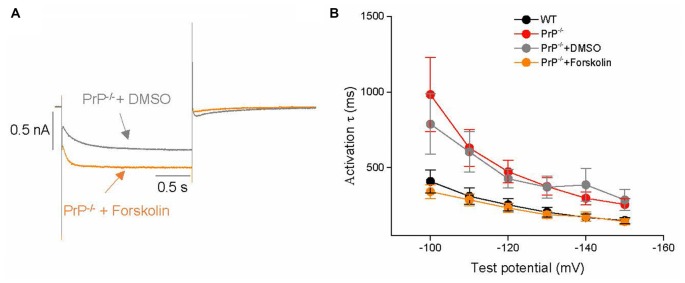
**Effects of forskolin on *I*_h_. (A)** Representative *I*_h_ current traces in the presence of forskolin or 0.1% DMSO (vehicle control) in PrP-null neurons. **(B)** Mean time constants of activation during hyperpolarizing steps from −150 to −100 mV after incubation with forskolin (*n* = 15) or vehicle (DMSO, *n* = 7) in PrP-null neurons. The slower kinetics in PrP-null neurons are reversed in the presence of forskolin.

One possible explanation for the reduction of *I*_h_ in PrP-null neurons is a PrP-mediated molecular signaling pathway that may downregulate HCN activity in the hippocampus. A prime candidate is the cAMP-dependent pathway, which is known to potently modulate HCN activity (Ingram and Williams, 1996; Wainger et al., 2001). This possibility was tested here by application of the adenylyl cyclase activator, forskolin (20 μM) on to PrP-null neurons (Figure 5A). Kinetic analysis showed that the slow activation kinetics could be reversed after a 10 min forskolin incubation in PrP-null neurons at voltages between −150 and −100 mV (Figure 5B). On the other hand, forskolin application did not modify *G*_max_ or half-activation voltage (*V*_0.5_: −82.1 ± 3.0 mV for PrP^−/−^ with DMSO (*n* = 7) vs. −87.4 ± 2.5 mV for PrP^−/−^ with Forskolin (*n* = 15), *p* > 0.05) in PrP-null neurons. Therefore, a downregulation of basal cAMP-dependent signaling in PrP-null neurons accounts for the slow *I*_h_ kinetics observed in PrP-null neurons, whereas the effect of PrP^C^ on HCN current amplitude and voltage-dependence of activation may be cAMP-independent.

## Discussion

### Modulation of Intrinsic Excitability of Hippocampal Neurons by PrP^C^

The principal finding of this study is that intrinsic excitability in neonatal cultured hippocampal neurons is regulated by PrP^C^, and that altered HCN channel activity appears to contribute to this effect. Specifically, an alteration of HCN channels is supported by differences in the voltage sag ratio between WT and null mouse neurons, and by the observation that the difference in input resistance was abolished upon application of the HCN channel blocker ZD7288. However, we cannot rule out the possibility that other ionic conductances might also be altered in response to ablation of PrP^C^ and may contribute to the overall increase in excitability. This could potentially include calcium activated potassium channels (Powell et al., 2008) and A-type potassium channels (Mercer et al., 2013), both of which have been shown to be regulated by PrP^C^. There is growing evidence that the loss of PrP^C^ function alters neuronal output, and therefore contributes to synchronized activities underlying neocortical and hippocampal seizures (Walz et al., 2002). Indeed, there are a number of electrophysiological and morphological abnormalities in hippocampal neurons from PrP-null mice (Collinge et al., 1994; Colling et al., 1996, 1997). First, an abnormal GABAAR inhibition has been reported in PrP-null mice (Colling et al., 1996) that may contribute to increased network excitability and, consequently, epileptiform activity. Second, PrP-null mice display aberrant sprouting of mossy fibers which is similar to the sprouting induced by seizures (Colling et al., 1997), indicating that this reorganization of neuronal circuitry may contribute an “epileptic neuronal network”. Third, a reduction in slow afterhyperpolarization currents (*I*_AHP_), which is of great importance for neuronal excitability, is evident in PrP-null mice (Colling et al., 1996; Herms et al., 2001; Mallucci et al., 2002; Fuhrmann et al., 2006). A recent study led to a new theory that neither K^+^ channels nor voltage-gated Ca^2+^ channels were responsible for this disrupted *I*_AHP_ in PrP-null CA1 pyramidal neurons (Powell et al., 2008). This was subsequently attributed to alterations in intracellular calcium buffering and clearance, which indirectly causes changes in potassium channel activity. Finally, a previous study from our lab has established a functional link between PrP^C^ and NMDARs, suggesting that PrP-null mice display an enhanced basal excitability due to enhanced NMDAR function (Khosravani et al., 2008).

It is however worth noting that many factors appear to affect what is observed in PrP-null mouse neurons, including the specific null mouse strain and age of the animals (Steele et al., 2007). Indeed, when we conducted experiments in hippocampal slices from adult mice, we did not observe differences in intrinsic excitability between WT and null mice, which might perhaps be due to age dependent changes in the regulation of HCN channels by PrP^C^. Further work will be required to determine whether there are indeed age dependent effects, or whether the observed differences are due to the different preparations used. Indeed, it is important to acknowledge potential limitations of our culture work. First, neurons in culture are isolated from neonatal animals and grown in artificial culture medium supplemented with exogenous growth factors for approximately 2 weeks. These conditions may result in changes in ion channel and receptor expression that could be different from those during normal postnatal development *in vivo*, and it is unclear that to what extent neurons grown in culture correspond to a specific age of neurons in an *in vivo* situation. Second, although we are confident that we were recording from pyramidal cells rather than interneurons, we cannot distinguish whether the cells were derived from the CA1 or CA3 regions and it is possible that cells from these two regions may express different levels of HCN channels. In contrast, in our slice recordings, we focused exclusively in CA1 pyramidal cells. Altogether, for future studies, it will be interesting to compare neuronal excitability in slices from younger (i.e., 2 week old) animals, and examining the properties of CA3 neurons in this preparation. Nonetheless, there appear to be multiple ways by which the absence of PrP^C^ can alter neuronal cell output that impacts network function, and at least in cultured neurons isolated from neonatal pups, PrP^C^ dependent regulation of HCN channels may be a contributing factor.

### Modulation of HCN Channel Activity by PrP^C^

HCN channels are highly expressed in the central nervous system and their function is critically related to neuronal excitability at both single-cell and network levels, particularly in the hippocampus (Robinson and Siegelbaum, 2003). HCN channels form heterotetramers or homotetramers, composed of HCN1–4 subunits; however, only HCN1 and HCN2 are highly expressed in hippocampus (Santoro et al., 2000), indicating a location-correlated function. They give rise to the *I*_h_ current, which we found to be downregulated in the absence of PrP^C^. HCN channels have unique biophysical properties that allow them to be partially active at rest and to act like a physiological “voltage clamp”. The decreased HCN current in cultured PrP-null mouse neurons is thus well positioned to account for the observed increase in input resistance. Moreover, a corresponding decrease in *I*_inst_ may amplify input resistance, thereby further increasing neuronal excitability. Whether the reduced *I*_h_ may also contribute to the well-established alterations of the *I*_AHP_ in PrP-null neurons remains to be tested. It is interesting to note that the decrease in *I*_h_ was not correlated with any apparent change in total or cell surface HCN protein expression, nor did there appear to be an HCN channel subunit switch. Furthermore, we note that our biochemical measurements were performed from hippocampal homogenate from adult mice, whereas recordings were done in cultured neurons from P0 pups. Given that the functional regulation of HCN channels by PrP^C^ appeared to be absent in slices from 2 month old mice, the biochemical analysis presented in Figure 4 does not rule out the possibility that there could be PrP^C^ dependent changes in HCN channel expression in our cultures. Nonetheless, the observation that HCN kinetics were altered in PrP-null neurons suggests that PrP^C^ is certainly capable of regulating the functional properties of HCN channels in native cells.

Our co-IP results that PrP^C^ did not form a complex with HCN suggest that HCN channel activity is not directly modulated by PrP^C^. It is well known that cAMP can directly bind to a cyclic-nucleotide domain in the C-terminal of the HCN channel (DiFrancesco and Tortora, 1991) and has been reported to shift the voltage dependence of HCN2 channel opening (Wainger et al., 2001); this in turn leads to an enhancement of *I*_h_ (Ingram and Williams, 1996). Although basal activities of cAMP have been observed to be enhanced in retinal tissue of PrP-null mice (Chiarini et al., 2002; Zanata et al., 2002; Lopes et al., 2005), the cAMP levels in hippocampal neurons of PrP-null mice remain unknown. Activation of cAMP affects numerous intracellular signaling cascades, including the activation of p38 mitogen-activated protein kinase (p38 MAPK) via the MAPK pathway (Gerits et al., 2008). Importantly, these enzymes are well recognized players in the regulation of neuronal excitability. In addition, the inhibition of p38 MAPK has been shown to induce a hyperpolarizing shift in *I*_h_ voltage-dependent activation and an increased input resistance (Poolos et al., 2006; Jung et al., 2010). In particular, the level of p38 MAPK appears to be downregulated in PrP-null mice, which may explain a hyperpolarizing shift in *I*_h_ gating and an increased input resistance in PrP-null mice observed in our study. Bath applied forskolin in PrP-null neurons was only able to rescue the kinetics from the slow state, whereas other parameters were unaffected. This then suggests that other signaling pathways also contribute to the PrP^C^-mediated regulation of HCN channels. Further experimentation will be required to delineate these signaling cascades.

In summary, our data suggest a regulatory role of PrP^C^ on HCN channel activity at both the cellular and molecular levels in cultured neurons, and suggests that PrP^C^ is coupled to signaling cascades that can converge on HCN channels.

## Author Contributions

JF, PLS, and GWZ designed the study. JF and GWZ wrote the manuscript. JF, SAB, IAS, MAG, ZZ and LC performed experiments. JF and MAG did data analysis. GWZ supervised the study.

## Conflict of Interest Statement

The authors declare that the research was conducted in the absence of any commercial or financial relationships that could be construed as a potential conflict of interest.
